# The value of evaluating cardiac damage in patients with aortic stenosis: a systematic review and meta-analysis

**DOI:** 10.1186/s44156-025-00089-w

**Published:** 2025-09-30

**Authors:** Sadie Bennett, Eric Holroyd, Maria F. Paton, Paul Leeson, Bjorn Redfors, Philippe Pibarot, Philippe Généreux, Chun Shing Kwok

**Affiliations:** 1 Heart and Lung Clinic, University Hospital of North Midlands NHS Trust, Stoke-on-Trent, UK; 2 Cardiovascular Clinical Research Facility, University of Oxford, Oxford, UK; 3 Leeds Teaching Hospitals NHS Trust, Leeds, UK; 4 Leeds Institute of Cardiovascular and Metabolic Medicine, School of Medicine, University of Leeds, Leeds, UK; 5 Oxford University Hospitals NHS Foundation Trust, John Radcliffe, Oxford, UK; 6 Department of Molecular and Clinical Medicine, Gothenburg University, Gothenburg, Sweden; 7 Department of Cardiology, Sahlgrenska University Hospital, Gothenburg, Sweden; 8 Clinical Trials Centre, Cardiovascular Research Foundation, New York City, USA; 9 Laval University, Quebec City, Canada; 10 Quebec Heart and Lung Institute, Quebec City, Canada; 11 Gagnon Cardiovascular Institute, Morristown Medical Center, Morristown, NJ USA; 12 Department of Cardiology, Mid Cheshire Hospitals NHS Foundation Trust, Crewe, UK

**Keywords:** Cardiac damage, Aortic stenosis, Systematic review

## Abstract

**Background:**

Aortic stenosis (AS) is a common valvular heart disease where aortic valve replacement (AVR) is the only treatment. A novel staging system based on cardiac damage was developed to assess the pathophysiological consequence of AS and this has been shown to be associated with outcomes post AVR.

**Methods:**

We conducted a systematic review of studies which evaluated cardiac damage in patients with AS. A search of MEDLINE and EMBASE was performed with data being extracted from relevant studies. The main outcome of interest were proportion of AS patients with signs of cardiac damage, all-cause mortality, cardiovascular mortality, and major adverse cardiovascular events.

**Results:**

A total of 18 studies were included with 21,876 patients (mean age 79 years, 52.7% males). Pooled analysis indicated 76% of symptomatic severe AS patients and 88% of asymptomatic moderate/severe AS patients had signs of cardiac damage, with stage two being the most commonly reported (25.1% and 32.3% respectively). For symptomatic severe AS patients, the pooled all-cause mortality and cardiovascular mortality rates increased along an increase in cardiac damage stage from 9.4% to 2.0% respectively for stage 0 to 24.2% and 36.1% respectively for stage 4. In patients with asymptomatic moderate / severe AS, all-cause mortality ranged from 30.0% in stage 0 to 51.2% in stage 3/4. In patients with symptomatic severe AS undergoing AVR, meta-analysis indicated an increase in odds of cardiovascular related mortality for stage 4 cardiac damage only (OR 6.89, 95% CI: 3.04,15.61, *p* = 0.003). An increased odds of all-cause mortality was seen in for cardiac damage stages 1, 3 and 4 (OR 1.4, 95%CI: 1.10,1.77, *p* = 0.01, OR 2.27, 95%CI: 1.76,2.92, *p* = 0.0002 and OR 2.94, 95%CI: 1.97,4.38, *p* = 0.0006 respectively).

**Conclusions:**

Cardiac damage is a common finding amongst patients with AS irrespective of AS severity or symptomatic status. Mortality rates appear to increase alongside an increase in cardiac damage staging. Cardiac damage may provide prognostic valve when considering the timing of AVR with left ventricular and right ventricular abnormalities being associated with increased odds of mortality.

**Clinical trial number:**

Not applicable.

**Supplementary Information:**

The online version contains supplementary material available at 10.1186/s44156-025-00089-w.

## Introduction

Degenerative aortic stenosis (AS) is a commonly acquired valvular heart disease condition in developed countries [1]. It is most prevalent among elderly patients with an incidence rate of 4.4% per year for patients aged ≥ 65 years [2]. In the early stages of AS, patients are typically asymptomatic however, as the condition progresses patients may develop symptoms including exertional dyspnoea, syncope and angina [3], subsequently resulting in poor quality of life [4] and high mortality rates [5]. The management of AS is limited to aortic valve replacement (AVR), with either surgical aortic valve replacement (SVAR) or transcatheter aortic valve replacement (TAVR) [6, 7]. 

**Fig. 1:**
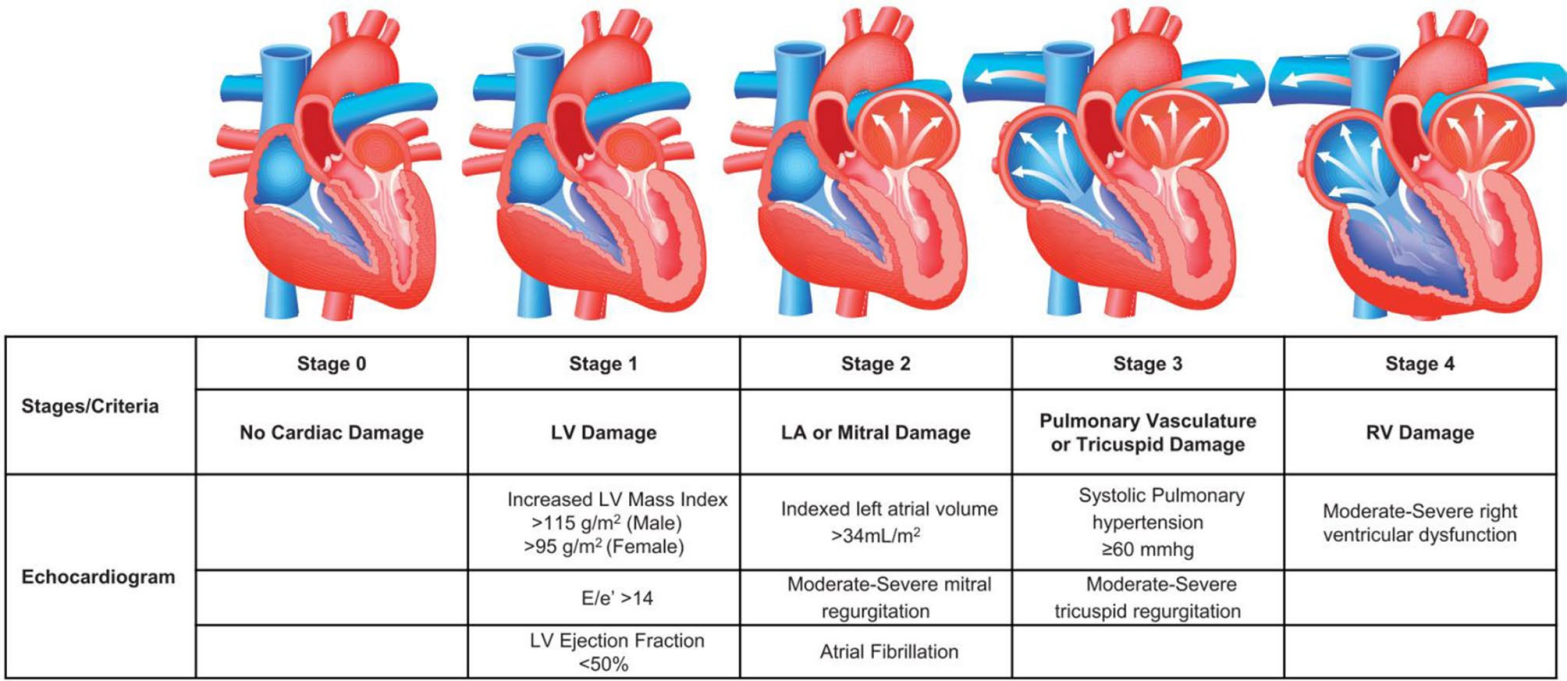
Cardiac damage staging classification. (From Généreux et al. EHJ 2017 with permission). The cardiac damage classification system was initially developed using transthoracic echocardiography and classified patients with severe AS into one of five stages. These stages were defined depending on the presence or absence of extra-valvular (extra-aortic) cardiac changes with or without dysfunction. The five stages (stage 0–4) include, stage 0; no cardiac damage, stage 1; left ventricular (LV) hypertrophy (LV mass index > 95 g/m^2^ for women, > 115 g/m^2^ for men), left ventricular diastolic dysfunction (E/e’ >14) or left ventricular systolic dysfunction (left ventricular ejection fraction < 50%), stage 2; left atrium enlargement (> 34 ml/m2), presence of atrial fibrillation, or presence of moderate or severe mitral regurgitation, stage 3; pulmonary vasculature damage (systolic pulmonary hypertension ≥ 60mmHg) or moderate to severe tricuspid regurgitation and stage 4: right ventricular dysfunction

Risk stratification for AS patients that require a AVR is limited and often based upon the presence of comorbidities only [6, 7]. In an attempt to improve the risk stratification and prognosis prediction for patients requiring AVR, Généreux et al., developed the concept of cardiac damage (see Fig. 1) to assess and classify anatomical and functional consequences of the increased loading conditions that occur a direct result of AS [8]. Since its development, it has been shown to have a strong association mortality in patients with severe AS undergoing TAVR [9, 10], along with patients with asymptomatic moderate to severe AS undergoing AVR [11]. 

We conducted a systematic review with the aims to assess the proportion of patients with severe AS who are classified into the different cardiac damage stages. We also aimed to assess the extent to which cardiac damage is associated with all-cause mortality, cardiovascular related mortality and major adverse cardiovascular events (MACE) in patients with severe AS.

## Methods

### Protocol registration

This review was registered with PROSPERO [CRD42023406744] and complies with the reporting guidelines and recommendations of MOOSE [12]. 

### Search strategy and eligibility criteria

A search was performed by CSK and SB on OVID of the databases MEDLINE and EMBASE and Web of Science to identify all study types evaluating cardiac damage in patients with severe AS. Electronic searches were complemented by manually searching all reference lists of identified studies and reviews for additional studies. The exact search terms were: (cardiac damage) AND (aortic stenosis OR aortic valve stenosis). The following MeSH terms were used across all databases: “aortic stenos$”, “cardiac damage”, “aortic valve stenos$”, “stenos$, aortic valve”, “stenos$, aortic”. There were no MeSH terms specific to cardiac damage. We include studies which evaluated cardiac damage in patients with severe AS irrespective of the underlying morphology of the aortic valve. There was no restriction based on language of publication, study design or date of publication.

One reviewer (CSK) conducted the initial search. Two reviewers (CSK and SB) independently screened all articles by title and abstract. Two reviewers (CSK and SB) then read the full text of potentially eligible studies and decided on which studies to include. Discrepancies were resolved by discussion or by a third reviewer where needed. Study inclusion criteria included: original reports that reported all-cause and/or cardiovascular mortality and MACE in patients who had severe AS and who were categorised as having cardiac damage stage 0, 1, 2, 3 or 4 based on transthoracic echocardiography. MACE was defined as acute myocardial infarction, stroke and hospitalisation for unstable angina, revascularisation procedures or heart failure [13]. We excluded studies where cardiac damage was not assessed, where it was likely that the same dataset was used within multiple studies and where no outcome data was reported for all-cause mortality, cardiovascular related mortality or MACE.

### Data extraction and risk of bias assessment

Data was collected independently by two reviewers (CSK and SB) using a standardised reporting form. The following information was extracted from eligible studies; cardiac damage classification, study design, follow-up, all-cause mortality and cardiovascular mortality, MACE and transthoracic echocardiographic (TTE) variables. Where multiple follow-up durations were reported, the longest follow-up duration which reported all required outcomes was used for data extraction. Disagreements between the two reviewers were resolved by discussion or by a third reviewer where needed. Study quality assessment was performed independently by CSK and SB using the Ottawa-Newcastle Scale [14]. Discrepancies were resolved by discussion or by a third reviewer where needed.

### Data synthesis and statistical analysis

The data collected were described in tables. We used RevMan 5.4 (Nordic Cochrane Centre) to perform random-effects meta-analysis using the inverse variance method to compare odds ratios for all-cause and cardiovascular mortality for patients with cardiac stage 1, 2, 3 and 4 vs. stage 0 for patients with symptomatic severe AS. For the meta-analysis, cardiac damage was assessed using TTE findings and based on the original Généreux et al. [8] cardiac damage classification method only. Data from studies were pooled where the crude rate of events and the total number of patients were reported by each cardiac damage staging group were available. Crude rates of events were converted into annualised event rates with 95% confidence intervals (CI) calculated via the poisson distribution methods using R (version 4.3.1) and R Studio (version 2024.04.0 + 735). Statistical heterogeneity was assessed using the I^2^ statistic where values of 30–60% represent a moderate degree of statistical heterogeneity. We intended to assess publication bias using Egger’s regression test and visualised with funnel plot, as appropriate given the known limitations of these methods [15].

## Results

### Risk of bias assessment

The study quality assessment is shown in the supplementary data, Table S1. All included studies demonstrated reliable selection of control groups, reliable ascertainment of exposure (cardiac damage stage), certainty that outcome was not present at the start of the study, reliable ascertainment of outcome (all-cause mortality and cardiovascular related mortality) and adequate follow-up duration. Eleven studies did not demonstrate good comparability [8–11, 16–22] and seven studies did not have adequacy of follow-up [8–10, 16, 18, 20, 21]. The average number of stars using the Ottawa-Newcastle scale was 7.4 out of 9 stars (range 6 to 9) indicating the quality of rating of the included studies to be good (intermediate risk of bias) or high quality (low risk of bias) in accordance with the Newcastle Ottawa Scale [14]. 

### Characteristics of included studies

**Fig. 2:**
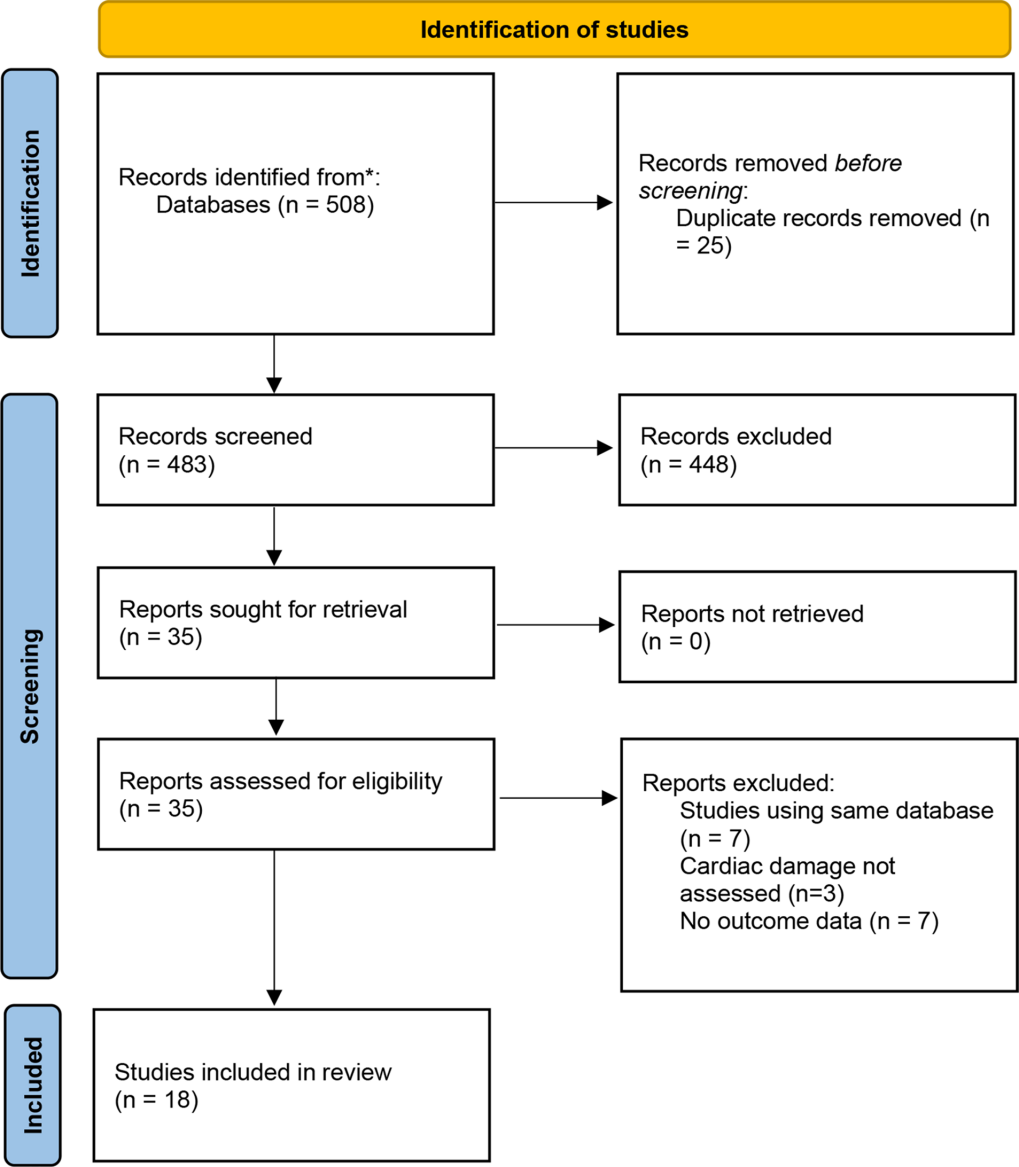
PRISMA flow diagram of study selection

The search was undertaken on 4th January 2024 and identified 508 potentially relevant records. After full text screening (see Fig. 2) we included a total of 18 studies [1, 8–11, 16–28]. A description of the study designs, participant characteristics, inclusion criteria can be seen in Table 1. All of the included studies were observation in study design, eight were prospective [1, 8–11, 16, 20, 21], and 10 retrospective in nature [17–19, 22, 24–28]. In total 21,867 patients were included of which, 50.1% (10,961/21,867) had symptomatic severe AS, 11.2% (2,452/21,864) had asymptomatic moderate / severe AS whilst the remaining patients consistent of severe AS with vary flow states (8,162/21,867, 37.6%) or had severe AS with acute decompensation (292/21,867, 1.3%). The average mean age across the studies was 79 years and the proportion of male patients was 52.7%. A description of the echocardiographic parameters for the included studies is shown in the supplementary data, Table S2. Only one study commented on aortic valve morphology and included patients who had a tricuspid or bicuspid aortic valve [14]. No studies reported excluding patients with a bicuspid/unicuspid valve. Follow up ranged from 30 days to 8 years. In studies where patients underwent AVR (TAVR or SAVR), the follow up period was from the intervention onwards. The cardiac damage classification, study population and outcomes of interest are shown in Table 2.

**Table 1:** Study design and participant characteristics

Study ID	Study design; Country; Year	Total population	Mean age	% Male	Participant inclusion criteria
Avvedimento 2021 [1]	Prospective observational study; single centre; Italy; 2014 to 2019.	262	79.8	38.2	Severe symptomatic AS undergoing TAVR in the EffecTAVR registry.
Belmonte 2024 [11]	Prospective observational study; single centre; Belgium; 2017 to 2021.	432	74.6	61.7	Moderate or severe AS who underwent right heart catheterisation within one month of TTE.
Berkovitch 2020 [23]	Retrospective observational study; multi-centre; Israel; 2008 to 2018.	2608	82.0	45.4	Severe symptomatic AS undergoing TAVR.
Fukui 2020 [24]	Retrospective observational study; unclear number of centres; United States; unclear years.	689	82.4	50.9	Severe symptomatic AS undergoing TAVR.
Généreux 2017 [8]	Prospective observational study; multicentre; International; 2011 to 2017.	1661	83.1	53.2	Severe symptomatic AS undergoing surgical AVR or TAVR in the PARTNER 2 trials.
Gutierrez-Ortiz 2023 [21]	Prospective observational study; single centre; Spain; 2017 to 2021.	496	82.1	47.0	Severe symptomatic AS undergoing TAVR.
Maeder 2020 [26]	Retrospective cohort study; single centre; Switzerland; 2011 to 2016.	421	74.6	58.9	Severe symptomatic AS undergoing SAVR or TAVR.
Okuno 2021 [16]	Prospective observational study; single centre; Switzerland; 2007 to 2016.	1133	82.1	49.2	Severe symptomatic AS undergoing TAVR.
Patel 2022 [27]	Retrospective observational study; single centre; United Kingdom; 2015 to 2019.	292	84	54.1	AS with acute decompensation undergoing TAVR.
Pellegrini 2022 [10]	Prospective observational study; single centre; Germany; 2011 to 2016.	841	81.1	52.7	Severe symptomatic AS undergoing TAVR.
Schewel 2021 [9]	Prospective observational study; single centre; Germany; 2008 to 2017.	1400	81.5	46.3	Severe symptomatic AS undergoing TAVR.
Sevilla 2023 [17]	Retrospective cohort study; single centre; Spain; unclear.	96	71	71.9	Moderate and severe asymptomatic AS who underwent CMR.
Shamekhi 2022 [22]	Retrospective observational study; two centre; Germany; 2008 to 2019.	933	80.5	50.3	Severe symptomatic AS undergoing TAVR.
Snir 2021 [20]	Prospective national registry; multicentre; Australia; 2000 to 2019.	8162	75.3	53.5	High gradient severe AS, classical low flow low gradient severe AS, paradoxical low gradient low gradient severe AS and normal flow low gradient severe AS on echocardiography.
Tastet 2019 [25]	Retrospective observational study; multicentre; International; 1998 to 2017.	735	71	60.1	Moderate to severe asymptomatic AS in a multi-centre registry.
Viva 2023 [18]	Retrospective cohort study; single centre; Belgium; 2016 to 2020.	90	82.2	44.4	Severe symptomatic AS undergoing TAVR.
Vollema 2019 [19]	Retrospective cohort study; multicentre; Netherlands and Singapore; 1999 to 2017.	1189	73.4	52.5	Moderate and severe asymptomatic AS referred for CMR evaluation.
Zhu 2022 [28]	Retrospective cohort study; single centre; China; 2013 to 2019.	427	76.1	58.3	Symptomatic severe AS undergoing TAVR.

AS; Aortic stenosis, CMR; cardiac magnetic resonance, SAVR; Surgical aortic valve replacement, TAVR; Transcatheter aortic valve replacement, TTE; Transthoracic echocardiogram

**Table 2:** Cardiac damage classification, population, follow-up, new York heart association classification and outcomes

Study ID	Cardiac damage classification used	Population	Follow-up	NYHA class	All-cause mortality	Cardiovascular mortality
Avvedimento 2021 [1]	Généreux classification with modification of left ventricular global longitudinal strain to stage 1	Severe symptomatic AS undergoing TAVR	1 year	I or II Stage 0/1: 18 Stage 2: 48 Stage 3: 25 Stage 4: 20 III or IV: Stage 0/1: 5 Stage 2: 58 Stage 3: 34 Stage 4: 54	Stage 0/1: 1/23 Stage 2: 7/106 Stage 3: 10/59 Stage 4: 15/74 HR vs. stage 0/1: Stage 2 h 1.55,95%CI 0.19–12.64, Stage 3 h 4.5,95% CI 0.58–35.07, stage 4: HR 5.49, 95% CI0.73-41.43 Cardiac damage and mortality: HR 1.745, 95% CI 1.17–2.60, *p* = 0.006	Stage 0/1: 1/23 Stage 2: 5/106 Stage 3: 7/59 Stage 4: 11/74
Belmonte 2024 [11]	Généreux classification	Moderate asymptomatic AS and asymptomatic severe AS	3 years	Severe symptomatic AS: Stage 0: NYHA I: 4/23 NYHA II: 15/23 NYHA III: 3/23 NYHA IV: 0/23 Stage 1: NYHA I: 5/53 NYHA II: 29/53 NYHA III: 7/53 NYHA IV: 3/53 Stage 2: NYHA I: 2/99 NYHA II: 53/99 NYHA III:29/99 NYHA IV:2/99 Stage 3: NYHA I: 0/38 NYHA II: 24/38 NYHA III: 12/38 NYHA IV: 1/38 Stage 4: NYHA I: 1/36 NYHA II:14/36 NYHA III: 14/36 NYHA IV: 3/36	Moderate/asymptomatic severe AS: Stage 0: 0/19 Stage 1: 2/46 Stage 2: 4/61 Stage 3: 4/30 Stage 4: 3/27 Severe symptomatic AS: Stage 0: 2/23 Stage 1: 14/53 Stage 2: 25/99 Stage 3: 17/38 Stage 4: 17/36	Moderate asymptomatic AS: Stage 0: 0/19 Stage 1: 0/46 Stage 2: 6/61 Stage 3: 4/30 Stage 4: 5/27 Severe symptomatic AS: Stage 0: 1/23 Stage 1: 3/53 Stage 2: 14/99 Stage 3: 12/38 Stage 4: 13/36
Berkovitch 2020 [23]	Généreux classification	Severe symptomatic AS undergoing TAVR	1 year	NYHA III/IV: Stage 0: 593/758 Stage 1: 598/769 Stage 2: 599/730 Stage 3: 262/320 Stage 4: 27/31	Stage 0: 52/758 Stage 1: 77/769 Stage 2: 88/730 Stage 3: 59/320 Stage 4: 9/31 Stage (by 1-point increment): HR 1.37 95%CI 1.23–1.54, p = < 0.001	Not reported
Zhu 2022 [28]	Généreux classification	Severe symptomatic AS undergoing TAVR	2 years	NYHA ≥ 3: Stage 1: 69/93 Stage 2: 338/426 Stage 3: 116/142 Stage 4: 20/28	Stage 0: Not reported Stage 1: 57/93 Stage 2: 241/426 Stage 3: 93/142 Stage 4: 17/28 HR vs. Stage 1 (reference): Stage 2 h 1.52 95% CI 0.89–2.56, *p* = 0.118 Stage 3 h 2.68 95% CI 1.54–4.67, *p* < 0.001 Stage 4 h 3.37 CI 95% 1.66–6.83, *p* < 0.001	Not reported
Généreux 2017 [8]	Généreux classification	Severe symptomatic AS undergoing SAVR or TAVR	1 year	Not reported	Stage 0: 2/47 Stage 1: 19/212 Stage 2: 116/844 Stage 3: 85/413 Stage 4: 33/145 Stage of cardiac damage (by each stage increase): HR 1.46 CI 95% 1.27–1.67, *p* < 0.001	Stage 0: 1/47 Stage 1: 15/212 Stage 2: 73/844 Stage 3: 48/413 Stage 4: 24/145
Gutierrez-Ortiz 2023 [21]	Généreux classification, Okunu classification and authors own classification	Severe symptomatic AS undergoing TAVR	1 year	NYHA ≥ 3: Stage 0/1: 27/58 Stage 2: 127/249 Stage 3: 30/58 Stage 4: 67/130	Author’s classification of cardiac damage Stage 0: 8/95 Stage 1: 29/166 Stage 2: 17/64 Stage 3: 18/63 No difference for death using the Genereux or Okunu classification.	Author’s classification of cardiac damage Stage 0: 1/95 Stage 1: 5/166 Stage 2: 3/64 Stage 3: 4/63 No difference for cardiovascular death using the Genereux or Okunu classification.
Maeder 2020 [26]	Généreux classification	Severe AS undergoing SAVR or TAVR	30 days and median of 3.8 years	Stage 0: NYHA I: 20/67 NYHA II: 33/67 NYHA III: 12/67 NYHA IV: 2/67 Stage 1: NYHA I: 30/113 NYHA II: 62/113 NYHA III: 19/113 NYHA IV: 2/113 Stage 2: NYHA I: 24/151 NYHA II: 74/151 NYHA III: 48/151 NYHA IV: 6/151 Stage 3: NYHA I: 7/73 NYHA II: 25/73 NYHA III: 31/73 NYHA IV: 10/73 Stage 4: NYHA I: 2/17 NYHA II: 7/17 NYHA III: 7/17 NYHA IV: 1/17	30 days follow-up: Stage 0: 4/67 Stage 1: 3/113 Stage 2: 2/151 Stage 3: 7/73 Stage 4: 2/17 Median 3.8 years follow-up: Stage 4 vs. stage 1: HR 6.17 95% CI 1.74–21.89, *p* = 0.005 Stage 3 vs. stage 0: HR 4.17 95% CI 1.39–12.49, *p* = 0.01 Stage 2 vs. stage 0: HR 0.98 95%CI 0.30–3.20, *p* = 0.98 Stage 1 vs. stage 0: HR 0.45 95% CI 0.10–20.3, *p* = 0.30	Not reported
Okuno 2021 [16]	Généreux classification	AS undergoing TAVR	1 year	NYHA III or IV: Stage 0 or 1: 88/151 Stage 2: 258/397 Stage 3: 167/239 Stage 4: 262/346	Généreux classification Stage 0: 2/38 Stage 1: 6/113 Stage 2: 35/397 Stage 3: 42/239 Stage 4: 88/346	Généreux classification Stage 0: 0/38 Stage 1: 2/113 Stage 2: 23/397 Stage 3: 30/239 Stage 4: 66/346
Patel 2022 [27]	Généreux classification	AS with acute decompensation undergoing TAVR	Mean of 2.4 years	Not reported	Stage 1: OR 1.621 95% CI 0.313–8.393, *p* = 0.565 Stage 2: OR 1.510 95% CI 0.365–6.330, *p* = 0.565 Stage 3: OR 3.023 95% CI 0.675–13.532, *p* = 0.148 Stage 4: OR 2.101 95% CI 0.510–8.655, *p* = 0.304	Not reported
Pellegrini 2022 [10]	Généreux classification	Severe symptomatic AS undergoing TAVR	2 years	NYHA III/IV: Stage 0: 2/7 Stage 1: 32/63 Stage 2: 346/532 Stage 3: 118/154 Stage 4: 70/85	Stage 0: 1/7 Stage 1: 7/63 Stage 2: 91/532 Stage 3: 39/154 Stage 4: 22/85	Not reported
Schewel 2021 [9]	Généreux classification	Severe symptomatic AS undergoing TAVR	4 years	NYHA ≥ III by invasive Stage 0: 118/138 Stage 1: 275/330 Stage 2: 416/469 Stage 3: 290/323 Stage 4: 126/140	Classification by echocardiography: Stage 0: 38/139 Stage 1: 70/187 Stage 2: 158/420 Stage 3: 182/351 Stage 4: 177/303 Classification by invasive measurement: Stage 0: 41/138 Stage 1: 134/330 Stage 2: 185/469 Stage 3: 176/323 Stage 4: 87/140	Classification by echocardiography: Stage 0: 4/139 Stage 1: 15/187 Stage 2: 29/420 Stage 3: 43/351 Stage 4: 72/303 Classification by invasive measurement: Stage 0: 6/138 Stage 1: 22/330 Stage 2: 41/469 Stage 3: 51/323 Stage 4: 36/140
Sevilla 2023 [17]	Généreux classification	Moderate and severe asymptomatic aortic stenosis	5 years	Not reported	Stage 0: 36/37 Stage 1: 26/32 Stage 2: 15/23 Stage 3/4: 3/4	Not reported
Shamekhi 2022 [22]	Généreux classification with modification	Severe symptomatic aortic stenosis	1 year	Not reported	Stage 0: 23/43 Stage 1: 85/160 Stage 2: 135/302 Stage 3: 39/90 Stage 4: 180/318	Not reported
Snir 2021 [20]	Généreux classification	High gradient AS, low flow low gradient AS, paradoxical low gradient low gradient AS and normal flow low gradient AS	1	Not reported	High gradient AS: Stage 0: 181/1695 Stage 1: 139/975 Stage 2: 316/2134 Stage 3/4: 250/797 Classic low flow low gradient AS: Stage 0: 0/0 Stage 1: 28/148 Stage 2: 80/256 Stage 3/4: 79/207 Paradoxical low flow low gradient: Stage 0: 27/328 Stage 1: 22/161 Stage 2: 46/256 Stage 3/4: 67/214 Normal flow low gradient AS: Stage 0: 20/259 Stage 1: 9/171 Stage 2: 50/406 Stage 3/4: 35/155	High gradient severe AS: Stage 0: 108/1695 Stage 1: 90/975 Stage 2: 211/2134 Stage 3/4: 186/797 Classic low flow low gradient severe AS: Stage 0: 0/0 Stage 1: 18/148 Stage 2: 51/256 Stage 3/4: 59/207 Paradoxical low flow low gradient severe S: Stage 0: 7/328 Stage 1: 6/161 Stage 2: 24/256 Stage 3/4: 31/214 Normal flow low gradient severe AS: Stage 0: 4/259 Stage 1: 4/171 Stage 2: 31/406 Stage 3/4: 20/155
Tastet 2019 [25]	Généreux classification with modification	Moderate to severe asymptomatic AS	8 years	NYHA I: Stage 0: 91/109 Stage 1: 160/195 Stage 2: 296/368 Stage 3: 14/16 Stage 4: 41/47	Généreux classification Stage 0: 20/109, (reference) Stage 1: 50/193, HR 2.10 95% CI 0.61–2.35, *p* = 0.59 Stage 2: 167/366, HR 2.27 95% CI 1.25–4.12, *p* = 0.007 Stage 3/4: 34/63, HR 3.16 95% CI 1.48–6.77, *p* = 0.003	Généreux classification Stage 0: 10,109, (reference) Stage 1: 29/193, HR 1.53 95% CI 0.57–4.17, *p* = 0.41 Stage 2: 93/366, HR 2.51 95% CI 1.00-6.30, *p* = 0.05 Stage 3/4: 31/63, HR 5.24 95% CI 1.79–15.40, *p* = 0.003
Viva 2023 [18]	Généreux classification	Severe symptomatic AS undergoing TAVR	Median 2.9 years	Not reported	Stage 0–2: 17/41 Stage 3: 5/10 Stage 4: 21/39	Stage 0–2: 5/41 Stage 3: 3/10 Stage 4: 11/39
Vollema 2019 [19]	Généreux classification	Moderate and severe asymptomatic AS	1 year	NYHA ≥ III: Stage 0: 27/97 Stage 1: 67/282 Stage 2: 189/588 Stage 3: 44/82 Stage 4: 66/104	1 year follow up: Stage 0: 7/97 Stage 1: 10/282 Stage 2: 13/588 Stage 3: 23/82 Stage 4: 34/140 Unclear longer follow up: Stage 0: 27/97 Stage 1: 32/282 Stage 2: 39/588 Stage 3: 55/82 Stage 4: 59/140	Not reported
Zhu 2022 [28]	Généreux classification	Biscuspid and tricuspid AS undergoing TAVR	1 month and 2 years	NYHA ≥ III: Stage 0: 160/199 Stage 1: 48/51 Stage 2: 65/67 Stage 3: 55/59 Stage 4: 49/51	Not reported	1 month Stage 0: 3/199 Stage 1: 1/51 Stage 2: 4/67 Stage 3: 3/59 Stage 4: 0/51 2-year mortality Stage 0: 90/199 Stage 1: 23/51 Stage 2: 32/67 Stage 3: 23/59 Stage 4: 38/51

AS: Aortic stenosis, LV: Left ventricle, NYHA: New York heart association classification, SAVR: Surgical aortic valve replacement, TAVR: Transcatheter aortic valve replacement

### Cardiac damage classification system

In studies with symptomatic severe AS patients, cardiac damage was assessed using Généreux original classification in nine studies [8–11, 16, 18, 23, 26, 28], in the remaining studies a modified cardiac damage classification system was implemented. In Avvedimento et al., stage 0 and 1 were combined into one stage (labelled stage 0/1, *n* = 23). In addition to this, left ventricular global longitudinal strain (LV-GLS) was incorporated into stage 1 to assist the detection of subclinical left ventricular systolic impairment [1]. In Fukui et al., patients with severe AS and cardiac damage stage 0 were omitted from the study [24]. In Shamekhi et al., the cardiac damage classification system was simplified into three stages including stage 0 (no cardiac damage), stage 1 (left ventricular ejection fraction < 50%, increased indexed left ventricular mass of > 95 g/m2 in females or > 115 g/m2 in males) and stage 2 (systolic pulmonary pressure ≥ 60mmHg, moderate to severe tricuspid regurgitation, TAPSE < 20mm) [22]. In Gutierrez-Ortiz et al., the cardiac damage classification was re-defined into 4 stages and included stage 0 (no cardiac damage), stage 1 (left-sided subclinical damage derived from left ventricular GLS ≥ 17%), stage 2 (left sided damage defined as moderate or severe mitral regurgitation) and stage 3 (right sided damage defined as right ventricular-arterial coupling < 0.35) [21].

In studies which included asymptomatic moderate or severe AS patients [17, 19, 25], cardiac damage was assessed using Généreux et al., original classification however two studies combined cardiac damage stages 3 and 4 [17, 25]. In the studies which included severe AS with acute decompensation [27] or severe AS with varying flow states [20], the original Généreux et al. classification was used with no modifications.

### Patients with symptomatic severe AS

In studies which evaluated symptomatic severe AS patients (*n* = 10,961), 12.0% (1,316/10,961) were classified as stage 0, 15.7% (1,721/10,961) as stage 1, 32.3% (3,542/10,961) as stage 2, and 15.8% (1,737/10,961) and 12.2% (1,332/10,961) as stage 3 and 4 respectively. From five studies [8, 9, 11, 16, 28], the pooled cardiovascular related mortality rates according to cardiac damage classification was 2.0% for stage 0 (9/446), 9.4% for stage 1 (58/616), 9.4% for stage 2 (171/1,827), 14.2% for stage 3 (156/1,1100) and 24.2% for stage 4 (213/881). The pooled all-cause mortality rates according to cardiac damage classification was 9.4% for stage 0 (101/1,079), 13.0% for stage 1 (196/1,510), 16.2% for stage 2 (515/3,173), 27.1% for stage 3 (430/1,588) and 36.1% for stage 4 (348/963). Tables 3 and 4 highlights the absolute and annualised events rates for all-cause mortality and cardiovascular related mortality respectively.

**Table 3:** Absolute and annualised all-cause mortality rates for patients with severe symptomatic AS

Study ID	Follow-up duration (years)	Absolute event rate per cardiac damage stage					Annualised event rates per cardiac damage stage (95% CI*)				
		0	1	2	3	4	0	1	2	3	4
Belmonte 2024 [11]	3	2	14	25	17	17	0.67 (0.1,2.4)	4.67 (2.55,7.83)	8.33 (5.39,12.30)	5.6 (3.30–9.07)	5.6 (3.30–9.07)
Berkovitch 2020 [23]	1	52	77	88	59	9	52 (38.8,61.2)	77 (60.8,96.2)	88 (70.6,108.4)	59 (44.9,76.1)	9 (4.1–17.1)
Généreux 2017 [8]	1	2	19	116	84	33	2 (0.2,7.2)	19 (11.4,29.7)	116 (95.9,139.1)	84 (67.0,103.9)	33 (22.7,46.3)
Maeder 2020 [26]	30 days	4	3	2	7	2	48 (35.4,63.6)	36 (25.2,49.8)	24 (15.4,35.7)	85 (67.9,105.1)	24 (15.4,35.7)
Okuno 2021 [16]	1	2	6	35	42	88	2 (0.1,7.2)	6 (2.2,13.1)	35 (24.4,48.7)	42 (30.3,56.8)	88 (70.6,108.4)
Pellegrini 2022 [10]	2	1	7	91	39	22	0.5 (0.01,2.8)	3.5 (1.4,7.2)	45.5 (36.6,55.9)	19.5 (13.9,26.7)	11 (6.9,16.7)
Schewel 2021 [9]	2	38	70	158	182	177	19 (13.4,26.1)	35 (27.3,44.2)	79 (67.2,92.3)	91 (78.3,105.2)	88.5 (75.9,102.5)

*Confidence internal derived by Poisson Distribution method

**Table 4:** Absolute and annualised cardiovascular related mortality rates for patients with severe symptomatic AS

Study ID	Follow-up duration (years)	Absolute event rate per cardiac damage stage					Annualised event rates per cardiac damage stage (95% CI*)				
		0	1	2	3	4	0	1	2	3	4
Belmonte 2024 [11]	3	1	3	14	12	13	0.3 (0.008,1.857)	1 (0.2,2.9)	4.6 (2.6,7.8)	4 (2.1,6.9)	4.3 (2.3,7.4)
Généreux 2017 [8]	1	1	15	73	48	24	1 (0.02,5.6)	15 (8.4,24.7)	73 (57.2,91.8)	48 (35.4,63.6)	24 (15.4,35.7)
Okuno 2021 [16]	1	0	2	23	30	66	0 (0.0,3.7)	2 (0.2,7.2)	23 (15.6,34.5)	30 (20.2,42.8)	66 (51.0,83.9)
Schewel 2021 [9]	2	4	15	29	43	72	2 (0.5,5.1)	7.5 (4.2,12.4)	14.5 (9.7,20.8)	21.5 (15.6,28.9)	36 (28.2,45.3)
Zhu 2022 [28]	2	90	23	32	23	38	45 (36.2,55.3)	11.5 (7.3,17.2)	16 (10.9,22.6)	11.5 (7.3,17.3)	19 (13.4,26.1)

*Confidence internal derived by Poisson Distribution method

**Fig. 3:**
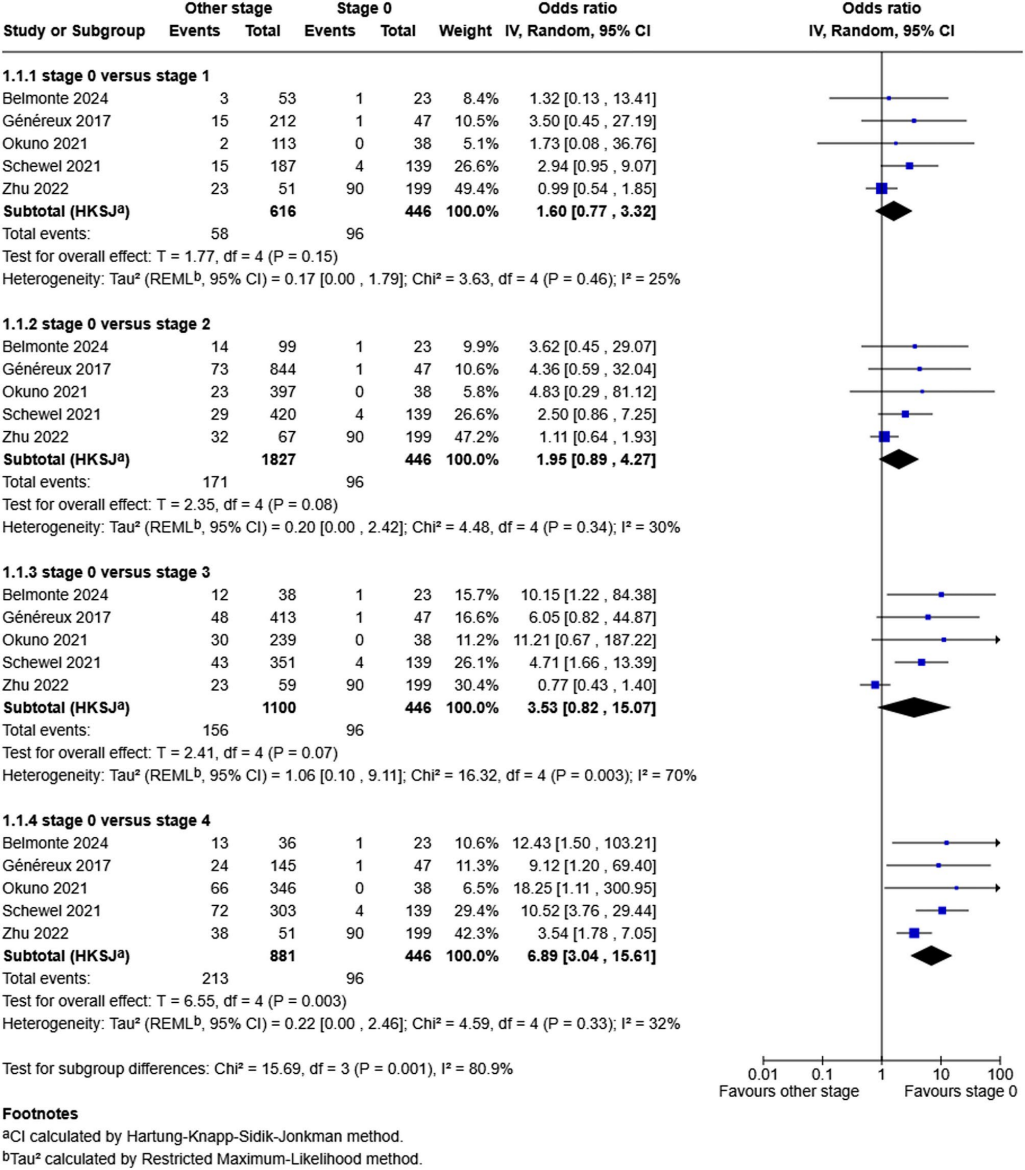
Meta-analysis evaluating the impact of cardiac stage on cardiovascular related mortality compared to stage 0 in patients with symptomatic severe AS

The meta-analysis from five studies [8, 9, 11, 16, 28] that evaluated cardiovascular related mortality in symptomatic severe AS patients is shown in Fig. 3. This indicates that when compared to cardiac damage stage 0, stage 1 and 2 was not associated with increased odds of cardiovascular related mortality (stage 1: OR 1.60, 95%CI: 0.77, 3.32, *p* = 0.15, stage 2: OR 1.95, 95% CI: 0.89, 4.27, *p* = 0.08 respectively) with no significant heterogeneity (*p* = 0.46, I^2^ = 25% and *p* = 0.34, I^2^ = 30% respectively). Stage 3, in comparison to stage 0, was not associated with an increased odds of mortality (OR 3.53, 95% CI: 0.82, 15.07, *p* = 0.07) however there was significant heterogeneity (*p* = 0.003, I^2^ = 71%) between the studies. When comparing stage 0 to stage 4, stage 4 was associated with 6-fold increased odds of cardiovascular mortality (OR 6.89, 95%CI: 3.04, 15.61, *p* = 0.003) with no significant heterogeneity (*p* = 0.33, I^2^ = 32%). Funnel plot testing for asymmetry was not performed due to the meta-analyses having fewer than 10 studies [15]. 

**Fig. 4:**
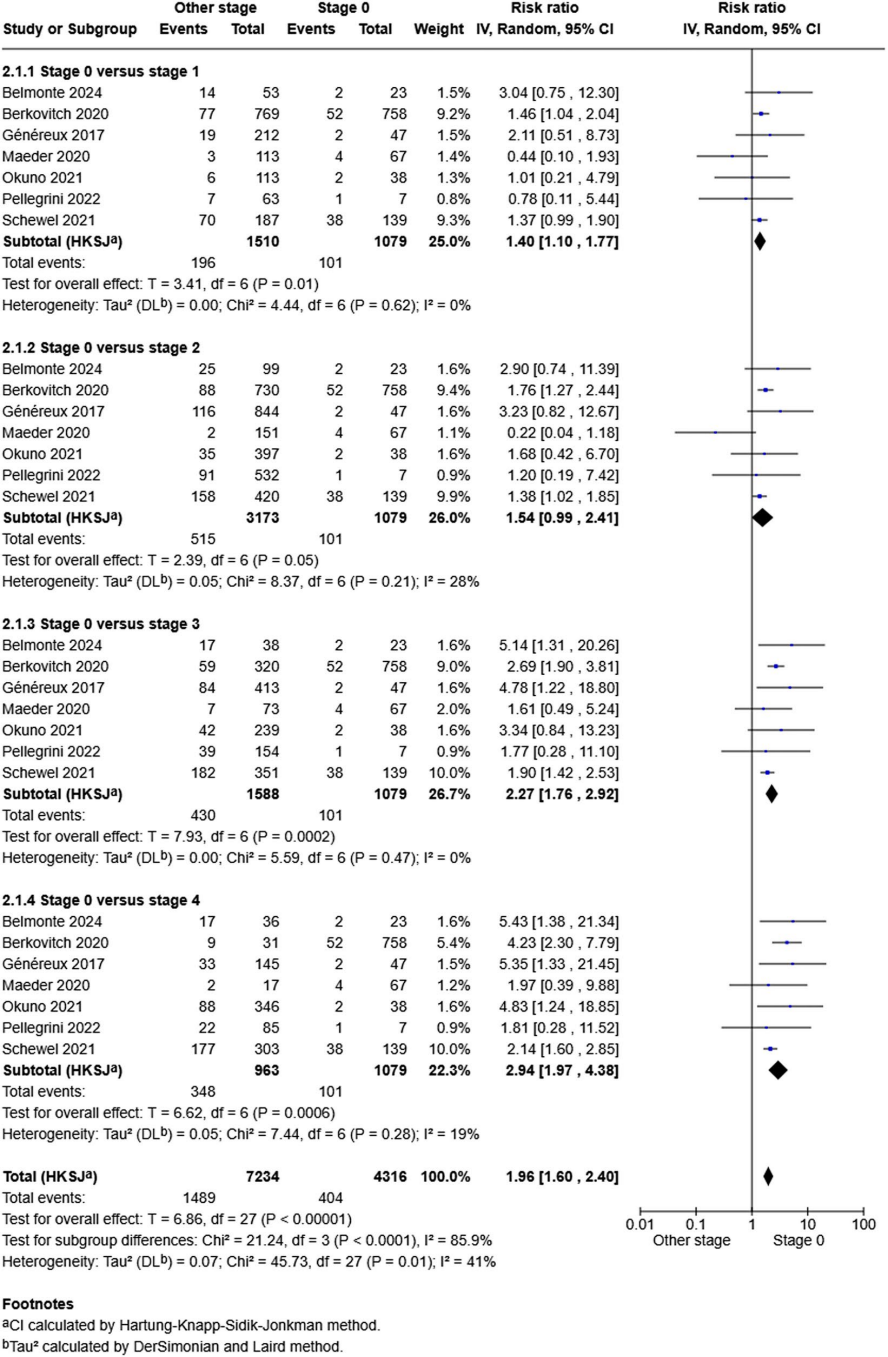
Meta-analysis evaluating the impact of cardiac stage on all-cause mortality compared to stage 0 in patients with symptomatic severe AS

Meta-analysis from seven studies [8–11, 16, 23, 26] that assessed all-cause mortality is shown in Fig. 4. This indicates that when compared to cardiac damage stage 0, stage 1 was associated with an increased odds of all-cause mortality (OR 1.4, 95%CI: 1.10, 1.77, *p* = 0.01) with no significant heterogeneity (*p* = 0.62, I^2^ = 0%). In comparison to stage 0, stage 2 was not associated with increased odds of all-cause mortality (OR 1.54, 95%CI: 0.99, 2.41, *p* = 0.05) with no significant heterogeneity (*p* = 0.21, I^2^ = 28%). When compared to stage 0, stage 3 and 4 were associated with increased odds of all-cause mortality (OR 2.27, 95%CI: 1.76, 2.92, *p* = 0.0002 and OR 2.94, 95%CI: 1.97, 4.38, *p* = 0.0006 respectively) with no significant heterogeneity (*p* = 0.47, I^2^ = 0% and *p* = 0.28, I^2^ = 19%). Funnel plot testing for asymmetry was not performed due to the meta-analyses having fewer than 10 studies [15].

### Patients with asymptomatic moderate or severe AS

In studies which investigated asymptomatic moderate or severe AS [19, 20, 24, 25] (*n* = 2,017), 12.0% (243/2,017) patients were classified into stage 0, 25.1% (507/2,017) stage 1, 48.5% (978/2,017) stage 2, and 14.3% (289/2,017) stage 3/4. The pooled rate of all-cause mortality for stage 0 was 30.0% (73/243), stage 1 was 17.2% (87/507), stage 2 was 15.0% (147/977) and stage 3/4 was 51.2% (148/289). One of these studies observed a significant increase in all-cause mortality and cardiovascular related mortality for stage 3 and 4 compared to stage 0 (HR 3.16 95%CI:1.48, 6.77 and HR 5.24 95%CI: 1.79, 15.40, respectively) [25]. 

### Other AS cohorts

The study by Snir et al. evaluated the impact of staging by cardiac damage in different subgroups of severe AS [20]. For all-cause mortality, the classic low-flow low-gradient severe AS cohort had the worse prognosis with mortality rates of 18.9% for stage 1, 31.3% for stage 2 and 38.2% for stage 3/4, while the severe AS group with normal flow low gradient had the lowest mortality (stage 0: 7.7%, stage 1: 5.3%, stage 2: 12.3%, and stage: 22.6%). In the high gradient severe AS cohort, mortality ranged from 10.7% for stage 0 to 31.4% for stage 3/4. In the paradoxical flow low gradient severe AS cohort, mortality ranged from 8.2% for stage 0 to 31.3% for stage 3/4. In the classic low flow low gradient severe AS cohort, mortality ranged from 0% for stage 1 to 38.1% for stage 3/4. For cardiovascular mortality, the highest percentage of deaths was observed for the classic low flow low gradient severe AS cohort (12.2% in stage 1, 19.9% in stage 2 and 28.5% in stage 3/4) and high-gradient severe AS cohort (6.4% in stage 0, 9.2% in stage 1, 9.9% in stage 2 and 23.3% in stage 3/4).

The study by Patel et al., evaluated patients with severe AS with acute decompensation [27]. At one year post TAVR, cardiac damage stage 2 and above independently predicted all-cause mortality (HR 1.85, 95%CI: 1.38, 3.85, *p* = 0.001). However, at 2.4 years (± 1.4 years), cardiac damage stage ≥ 2 was not associated with increased risk of all-cause mortality (HR 1.4, 95%CI: 0.93, 2.22, *p* = 0.098). Instead, at this extended follow-up time duration, only the clinical frailty score was associated with increased risk of all-cause mortality (HR: 1.66, 95%CI: 1.04, 2.66, *p* = 0.032 respectively).

### Major adverse cardiovascular events outcome for patients with AS

Where reported within the included studies, whilst there were generally low rates of MACE, there was a general trend for higher MACE incidence to occur in patients who were classified as having stage 3 or 4 cardiac damage, this was irrespective of whether patients had moderate or severe AS who underwent TAVR or SAVR. Full reporting of MACE outcomes can be seen in the supplementary data, Table S3.

## Discussion

This systematic review and meta-analysis sort to identify the proportion of AS patients who were classified into each of the cardiac damage stages in accordance with Généreux et al. [8], it also sort to investigate the extent to which each cardiac damage stage was associated with all-cause mortality, cardiovascular related mortality and MACE. The main findings are: (1) A large proportion of patients with AS, regardless of symptomatic status or AS severity, demonstrate evidence of cardiac damage at baseline. (2) The pooled and annualised all-cause and cardiovascular related mortality rates increase as the stage of cardiac damage increases. (3) Meta-analysis shows an increased odds for all-cause mortality for symptomatic severe AS patients with cardiac damage stages 1, 3 and 4. In addition to this, a 6-fold increase in odds of cardiovascular mortality was seen for symptomatic severe AS patients with cardiac damage stage 4. (4) The use of cardiac damage appears to have some prognostic value in asymptomatic moderate or severe AS however there is limited evidence to suggest this for patients with AS and varying flow states / acute decompensation.

Our pooled analysis indicated that 76.0% of patients with symptomatic severe AS and 88.0% of patients with asymptomatic moderate or severe AS had evidence of cardiac damage (cardiac damage stage ≥ 1) at baseline. Interestingly, in both patient groups, stage 2 cardiac damage was the most commonly reported stage (25.1% and 32.3% respectively). However, the cardiac damage classification system may overestimate the number of AS patients who have signs of cardiac damage and caution maybe required when classifying patients, particularly for cardiac damage stage 2 whereby a left atrial volume of > 34mL/msq is used to indicate the presence of left atrial / mitral damage. When considering the NORRE dataset a left atrial volume of ≥ 34mL/msq was noted to be present in 11% of patients with entirely normal hearts [29]. Therefore, the use of a left atrial volume of ≥ 34mL/msq value may increase the number of patients who are classified as having stage 2 cardiac damage who otherwise would be considered to have no signs of cardiac damage.

In patients with symptomatic severe AS, the pooled all-cause and cardiovascular related mortality rates increase with an increase in the cardiac damage staging. This finding remained for annualised event rates to account for differences in follow-up duration. However, from the meta-analysis only stages 1, 3 and 4 were associated with increased odds of all-cause mortality and only stage 4 was associated with increase odds of cardiovascular related mortality. These findings highlight the impact of the pathophysiological consequences of AS including left ventricular hypertrophy, Left ventricular systolic impairment, right ventricular impairment and pulmonary vascular remodelling [30]. Further supporting the notion that AS is a disease of the myocardium and not just the aortic valve [31]. Our meta-analysis indicates the importance of right heart abnormalities and their association with a 2-fold increase in the odds of all-cause mortality and 6-fold increase in the odds of cardiovascular related mortality. This is consistent with previous findings whereby right ventricular abnormalities in severe AS patients are associated with a higher risk of cardiovascular mortality at one year compared to patients with normal right ventricular function (adjusted hazard ratio: 2.94 95%CI 2.02, 4.27, *p* < 0.001) [32].

This review identifies that just over one third of patients with asymptomatic moderate or severe AS have features of cardiac damage and extends the notion that these patients have poor outcomes. Previous studies have shown that moderate AS patients have increased heart failure admissions [33, 34], and reduced survival rates, with a 5 year survival rates reported as low as 52% [35]. The main driver behind this is thought to include LV systolic impairment which can impair the LV’s ability to overcome increased afterload that results from AS [34]. Therefore, the use of cardiac damage may add value in determining the need for AVR in asymptomatic moderate / severe AS patients particularly as cardiac damage stages 3/4 is associated with an increased risk of mortality irrespective of the absence of symptoms [36]. The value of assessing cardiac damage in AS patients presenting late or, those with decompensation appears is clear as the current cardiac damage staging classification may oversimplify the complex AS pathophysiology seen and may not take into account patient’s comorbidities [24], which have been shown to be an independent predictor of all-cause mortality in this patient cohort [27]. 

Current class I indications for AVR include the development of symptoms or the presence of left ventricular systolic impairment, with a left ventricular ejection fraction (LVEF) < 50% [6, 37]. This indication for AVR currently sits within cardiac damage stage I which, as this review shows, is associated with an increased risk of all-cause mortality. However, previous research highlights that current AVR indications may be insensitive to subclinical LV systolic function as in the presence of LV hypertrophy, LVEF may be underestimated [36]. This is further impacted by previous literature indicated an LVEF of between 50 and 60% in AS patients being linked to increased MACE compared to an LVEF ≥ 60% [38]. Therefore, adjustments to the cardiac damage staging classification to include an LVEF ≥ 60%, or include LV-GLS could be considered to improve the prognostic value of the cardiac damage staging system.

Several authors have modified the original staging system in an attempt to improve the classification of extra-valvular cardiac damage. These modifications include the inclusion of additional echocardiographic variables such as LV-GLS, indexed stroke volume index and right ventricular atrial coupling. The prognostic relevant of these modifications and the impact on mortality was only assessed in one study [21]. Here, no difference in all-cause or cardiovascular related mortality was observed between the adapted and original cardiac damage classification system. Whilst there is promise in the original cardiac damage system, a simplified approach utilising echocardiography measurements that is able to detect early and subtle extra-cardiac damage abnormalities including LV-GLS and right ventricular coupling [39] may enable this approach to be more universally applied within clinical practice, whilst protecting the prognostic ability of staging system. Further studies are required to develop the cardiac damage system and its application within AS patients of varying symptomatic status and AS severity.

This review has several limitations. First, the data is derived from observational studies which may be influenced by confounders which could impact mortality outcomes. However, cardiac damage is not an exposure which it is possible to randomise patients with aortic stenosis to receive. Second, many of the cohort included patients with TAVR or SAVR and there may be selection biases that influence whether patients receive these treatments. Third, there was high heterogeneity between studies and there is insufficient evidence to assess the prognostic valve of cardiac damage stages 1 and 2. Four, many of the included studies follow up patients over short / mid-term time frames. It is not known whether cardiac damage persists and whether the final outcome is more influenced by the type of AVR, co-existing co-morbidities care received rather than the initial cardiac damage staging classification.

It should be noted that an additional systematic review on cardiac damage has recently been published by Abdelfattah et al. [40] Whilst our findings are generally consistent with the findings of this review, the studies included within both systematic reviews differs slightly. We choose to exclude two studies [41, 42] that were included within the review conducted by Abdelfattah et al. This was due to there being a high likelihood that the data presented within these studies was also included within Vollema et al. [19]. We therefore choose to include Vollema et al. [19] only as this was the larger dataset out of the three studies. It also ensured the included data was not duplicated, potentially creating bias within the results. Additionally, we choose to exclude Généreux et al. [43] as this article reported data on quality of life which was not the main focus of our review. The final discrepancy involves a study data that was presented within a letter [44], it was therefore excluded during the screening process. The data within the letter would not have altered the findings of our review as this study investigated asymptomatic patients with very severe AS.

## Conclusions

Cardiac damage is a common finding amongst patients with AS irrespective of AS severity or symptomatic status. Mortality rates appear to increase alongside an increase in cardiac damage staging. Cardiac damage may provide prognostic valve when considering the timing of AVR with left ventricular and right ventricular abnormalities being associated with increased odds of mortality.

## Electronic supplementary material

Below is the link to the electronic supplementary material.

**Supplementary Material 1**: Supplementary data, Table S1 Study quality assessment. Supplementary data, Table S2 Echocardiographic parameters. Supplementary data, Table S3 Cardiac damage classification, follow-up and major adverse cardiac event outcomes.

## Data Availability

No datasets were generated or analysed during the current study.

## Abbreviations

AS: Aortic Stenosis
AVR: Aortic Valve Replacement
CI: Confidence Interval
LA: Left Atrium
LAVi: Left Atrial Volume index
LV: Left Ventricle / Left Ventricular
LVEF: Left Ventricular Ejection Fraction
LV-GLS: Left Ventricular Global Longitudinal Strain
MACE: Major Adverse Cardiovascular Events
MOOSE: Meta-analysis of Observational Studies in Epidemiology
OR: Odds Ratio
PASP: Pulmonary Artery Systolic Pressures
RV: Right Ventricle / Right Ventricular
SAVR: Surgical Aortic Valve Replacement
TAPSE: Tricuspid Annular Systolic Excursion
TAVR: Transcatheter Aortic Valve Replacement
TTE: Transthoracic Echocardiographic

## References

[1] Avvedimento M, et al. Extent of cardiac damage and mortality in patients undergoing transcatheter aortic valve implantation. J Clin Med. 2021;10:4563. 34640580 10.3390/jcm10194563 PMC8509290

[2] Durko AP, et al. Annual number of candidates for transcatheter aortic valve implantation per country: current estimates and future projections. Eur Heart J. 2018;39:2635–42. 29546396 10.1093/eurheartj/ehy107

[3] Carabello BA, Paulus WJ. Aortic stenosis. Lancet. 2009;373:956–66. 19232707 10.1016/S0140-6736(09)60211-7

[4] Gracia Baena JM, et al. Impact of severe aortic stenosis on quality of life. PLoS ONE. 2023;18:e0287508. 37343035 10.1371/journal.pone.0287508 PMC10284408

[5] Iung B, Vahanian A. Degenerative calcific aortic stenosis: a natural history. Heart. 2012;98(Suppl 4):iv7–13. 23143128 10.1136/heartjnl-2012-302395

[6] Vahanian A et al. 2021 ESC/EACTS Guidelines for the management of valvular heart disease: Developed by the Task Force for the management of valvular heart disease of the European Society of Cardiology (ESC) and the European Association for Cardio-Thoracic Surgery (EACTS). *European Heart Journal* 43, 561–632 (2022). 10.1016/j.rec.2022.05.006 35636831

[7] Nishimura RA, Vahanian A, Eleid MF, Mack MJ. Mitral valve disease–current management and future challenges. Lancet. 2016;387:1324–34. 27025438 10.1016/S0140-6736(16)00558-4

[8] Généreux P, et al. Staging classification of aortic stenosis based on the extent of cardiac damage. Eur Heart J. 2017;38:3351–8. 29020232 10.1093/eurheartj/ehx381 PMC5837727

[9] Schewel J, Kuck K-H, Frerker C, Schmidt T, Schewel D. Outcome of aortic stenosis according to invasive cardiac damage staging after transcatheter aortic valve replacement. Clin Res Cardiol. 2021;110:699–710. 33744987 10.1007/s00392-021-01835-w

[10] Pellegrini C, et al. The impact of extra-valvular cardiac damage on mid-term clinical outcome following transcatheter aortic valve replacement in patients with severe aortic stenosis. Front Cardiovasc Med. 2022;9:1039208. 36531697 10.3389/fcvm.2022.1039208 PMC9751869

[11] Belmonte M, et al. Combined cardiac damage staging by echocardiography and cardiac catheterization in patients with clinically significant aortic stenosis. Can J Cardiol. 2024;40:643–54. 37979721 10.1016/j.cjca.2023.11.010

[12] Stroup DF, et al. Meta-analysis of observational studies in epidemiology: a proposal for reporting. Meta-analysis of observational studies in epidemiology (MOOSE) group. JAMA. 2000;283:2008–12. 10789670 10.1001/jama.283.15.2008

[13] Marx N, et al. Composite primary end points in cardiovascular outcomes trials involving type 2 diabetes patients: should unstable angina be included in the primary end point?? Diabetes Care. 2017;40:1144–51. 28830955 10.2337/dc17-0068

[14] Wells GA, Shea B, O’Connell D, Peterson J, Welch V, Losos M, Tugwell P. The Newcastle-Ottawa Scale (NOS) for assessing the quality if nonrandomized studies in meta-analyses, 2012. https://www.ohri.ca/programs/clinical_epidemiology/oxford.asp

[15] Higgins JT, Thomas J. Cochrane Handbook for Systematic Reviews of Interventions. Version 6.4, 2023. Available at: https://training.cochrane.org/handbook/current. Last accessed April 23 2024.

[16] Okuno T, et al. Staging cardiac damage associated with aortic stenosis in patients undergoing transcatheter aortic valve implantation. Int J Cardiol Heart Vasc. 2021;33:100768. 33898731 10.1016/j.ijcha.2021.100768 PMC8053801

[17] Sevilla T, et al. Staging cardiac damage and prognosis in asymptomatic aortic stenosis: early surgery might not benefit all. J Am Soc Echocardiogr. 2023;36:121–3. 36332802 10.1016/j.echo.2022.10.018

[18] Viva T, et al. A new integrative approach combining right heart catheterization and echocardiography to stage aortic stenosis-related cardiac damage. Front Cardiovasc Med. 2023;10:1184308. 37600042 10.3389/fcvm.2023.1184308 PMC10436206

[19] Vollema EM, et al. Staging cardiac damage in patients with symptomatic aortic valve stenosis. J Am Coll Cardiol. 2019;74:538–49. 31345429 10.1016/j.jacc.2019.05.048

[20] Snir AD, et al. Cardiac damage staging classification predicts prognosis in all the major subtypes of severe aortic stenosis: insights from the National echo database Australia. J Am Soc Echocardiogr. 2021;34:1137–e114713. 34082021 10.1016/j.echo.2021.05.017

[21] Gutierrez-Ortiz E, et al. Redefining cardiac damage staging in aortic stenosis: the value of GLS and RVAc. Eur Heart J Cardiovasc Imaging. 2023;24:1608–17. 37315235 10.1093/ehjci/jead140

[22] Shamekhi J, et al. A simplified cardiac damage staging predicts the outcome of patients undergoing TAVR-A multicenter analysis. Catheter Cardiovasc Interv. 2022;100:850–9. 35989489 10.1002/ccd.30368

[23] Berkovitch A, et al. Validation of cardiac damage classification and addition of albumin in a large cohort of patients undergoing transcatheter aortic valve replacement. Int J Cardiol. 2020;304:23–8. 32008849 10.1016/j.ijcard.2020.01.031

[24] Fukui M, Cavalcante JL. Effect of the Extent of Cardiac Damage on Transcatheter Aortic Valve Replacement Outcome: A New Aortic Stenosis Staging System. US Cardiol Rev. 2019;13(2):69–73. 10.15420/usc.2019.9.1

[25] Tastet L, et al. Staging cardiac damage in patients with asymptomatic aortic valve stenosis. J Am Coll Cardiol. 2019;74:550–63. 31345430 10.1016/j.jacc.2019.04.065

[26] Maeder MT, et al. Invasive hemodynamic staging classification of cardiac damage in patients with aortic stenosis undergoing valve replacement. Can J Cardiol. 2020;36;1667–1674. 10.1016/j.cjca.2020.02.004 32416065 10.1016/j.cjca.2020.02.004

[27] Patel KP, et al. Preprocedural prognostic factors in acute decompensated aortic stenosis. Am J Cardiol. 2022;174:96–100. 35527043 10.1016/j.amjcard.2022.03.037

[28] Zhu Q, et al. Validation of a novel staging classification system based on the extent of cardiac damage among Chinese patients after transcatheter aortic valve replacement: A single-center retrospective study. Catheter Cardiovasc Interv. 2022;99(Suppl 1):1482–9. 35324060 10.1002/ccd.30147

[29] Kou S, et al. Echocardiographic reference ranges for normal cardiac chamber size: results from the NORRE study. Eur Heart J Cardiovasc Imaging. 2014;15:680–90. 24451180 10.1093/ehjci/jet284 PMC4402333

[30] Bhattacharyya S, et al. Clinical and prognostic value of stress echocardiography appropriateness criteria for evaluation of coronary artery disease in a tertiary referral centre. Heart. 2014;100:370–4. 24310519 10.1136/heartjnl-2013-304949

[31] Dweck MR, Boon NA, Newby DE. Calcific aortic stenosis: A disease of the valve and the myocardium. J Am Coll Cardiol. 2012;60:1854–63. 23062541 10.1016/j.jacc.2012.02.093

[32] Asami M, et al. Prognostic value of right ventricular dysfunction on clinical outcomes after transcatheter aortic valve replacement. JACC Cardiovasc Imaging. 2019;12:577–87. 29454762 10.1016/j.jcmg.2017.12.015

[33] Banovic M, Iung B, Wojakowski W, Van Mieghem N, Bartunek J. Asymptomatic severe and moderate aortic stenosis: time for appraisal of treatment indications. Struct Heart. 2023;7:100201. 37745683 10.1016/j.shj.2023.100201 PMC10512009

[34] Coisne A, et al. Impact of moderate aortic stenosis on Long-Term clinical outcomes. JACC: Cardiovasc Interventions. 2022;15:1664–74. 10.1016/j.jcin.2022.06.022 35981841

[35] Du Y, et al. Natural history observations in moderate aortic stenosis. BMC Cardiovasc Disord. 2021;21:108. 33607944 10.1186/s12872-021-01901-1 PMC7893941

[36] Tastet L, Vincent F, Pibarot P. Cardiac damage staging in aortic stenosis: A perspective from the cardiac catheterization laboratory. Can J Cardiol. 2020;36:1583–6. 32634394 10.1016/j.cjca.2020.03.033

[37] Nishimura RA, et al. 2014 AHA/ACC guideline for the management of patients with valvular heart disease: a report of the American college of cardiology/american heart association task force on practice guidelines. J Am Coll Cardiol. 2014;63:e57–185. 24603191 10.1016/j.jacc.2014.02.536

[38] Capoulade R, et al. Echocardiographic predictors of outcomes in adults with aortic stenosis. Heart. 2016;102:934–42. 27048774 10.1136/heartjnl-2015-308742

[39] He Q, et al. Clinical usefulness of right Ventricle–Pulmonary artery coupling in cardiovascular disease. J Clin Med. 2023;12:2526. 37048609 10.3390/jcm12072526 PMC10095537

[40] Abdelfattah OM, et al. Cardiac damage staging predicts outcomes in aortic valve stenosis after aortic valve replacement: Meta-Analysis. JACC Adv. 2024;3:100959. 38939639 10.1016/j.jacadv.2024.100959 PMC11198616

[41] Hirasawa K, et al. Prognostic implications of cardiac damage classification based on computed tomography in severe aortic stenosis. Eur Heart J Cardiovasc Imaging. 2022;23:578–85. 33855450 10.1093/ehjci/jeab071

[42] Amanullah MR, et al. Prognostic implications of associated cardiac abnormalities detected on echocardiography in patients with moderate aortic stenosis. JACC Cardiovasc Imaging. 2021;14:1724–37. 34023268 10.1016/j.jcmg.2021.04.009

[43] Généreux P, et al. Evolution and prognostic impact of cardiac damage after aortic valve replacement. J Am Coll Cardiol. 2022;80:783–800. 35595203 10.1016/j.jacc.2022.05.006

[44] Park S-J, et al. Impact of early surgery and staging classification on survival in asymptomatic very severe aortic stenosis. J Am Coll Cardiol. 2021;77:506–8. 33509403 10.1016/j.jacc.2020.11.045

