# Supplementary material for: The value of evaluating cardiac damage in patients with aortic stenosis: a systematic review and meta-analysis
Source: Echo Res Pract. 2025 Sep 30;12:29. doi: 10.1186/s44156-025-00089-w (PMC12482353; doi:10.1186/s44156-025-00089-w)
Supplement: Supplementary file 1 — Supplementary Material 1: Supplementary data, Table S1 Study quality assessment. Supplementary data, Table S2 Echocardiographic parameters. Supplementary data, Table S3 Cardiac damage classification, follow-up and major adverse cardiac event outcomes. [file 44156_2025_89_MOESM1_ESM.docx]

**Supplementary data, *Table S1*:** Study quality assessment.

| **Study ID** | **Good representativeness of cohort (AS cohort)** | **Reliable selection of non-exposed cohort (Cardiac damage stage 0)** | **Reliable ascertainment of exposure (Cardiac damage stage >0)** | **Certainty that outcome present at start of study (mortality at baseline)** | **Good comparability (adjusted results presented)** | **Reliable outcome ascertainment (mortality ascertainment)** | **Follow up long enough for events (>1 year)** | **Adequacy of follow up (<10% not included)** | **Number of stars** |
| --- | --- | --- | --- | --- | --- | --- | --- | --- | --- |
| Avvedimento 2021 | Yes, severe symptomatic AS undergoing TAVR | Yes, reliable evaluation of cardiac damage | Yes, reliable evaluation of cardiac damage | Yes, no mortality | Yes, adjusted results | Yes, reliable ascertainment of mortality | Yes, 1 year | Yes, 13/275 patients not included | 9 |
| Belmonte 2023 | Yes, moderate or asymptomatic severe AS and symptomatic severe AS | Yes, reliable evaluation of cardiac damage | Yes, reliable evaluation of cardiac damage | Yes, no mortality | No, unadjusted results | Yes, reliable ascertainment of mortality | Yes, 3 years | Yes, missing data not reported | 7 |
| Berkovitch 2020 | Yes, severe symptomatic AS undergoing TAVR | Yes, reliable evaluation of cardiac damage | Yes, reliable evaluation of cardiac damage | Yes, no mortality | Yes, adjusted results | Yes, reliable ascertainment of mortality | Yes, 1 year | Yes, 80/2688 patients not included | 9 |
| Fukui 2020 | Yes, severe symptomatic AS undergoing TAVR | Yes, reliable evaluation of cardiac damage | Yes, reliable evaluation of cardiac damage | Yes, no mortality | Yes, adjusted results | Yes, reliable ascertainment of mortality | Yes, 2 years | Yes, missing data not reported | 9 |
| Généreux 2017 | Yes, severe symptomatic AS undergoing TAVR | Yes, reliable evaluation of cardiac damage | Yes, reliable evaluation of cardiac damage | Yes, no mortality | No, unadjusted results | Yes, reliable ascertainment of mortality | Yes, 1 year | No, 1042/2703 patients not included | 6 |
| Gutierrez-Ortiz 2023 | Yes, severe symptomatic AS undergoing TAVR | Yes, reliable evaluation of cardiac damage | Yes, reliable evaluation of cardiac damage | Yes, no mortality | No, unadjusted results | Yes, reliable ascertainment of mortality | Yes, 1 year | No, unclear as missing data was excluded | 6 |
| Maeder 2020 | Yes, severe symptomatic AS undergoing TAVR or SAVR | Yes, reliable evaluation of cardiac damage | Yes, reliable evaluation of cardiac damage | Yes, no mortality | Yes, adjusted results | Yes, reliable ascertainment of mortality | Yes, 30 days and median of 3.8 years | Yes, missing data not reported | 9 |
| Okuno 2021 | Yes, AS undergoing TAVR | Yes, reliable evaluation of cardiac damage | Yes, reliable evaluation of cardiac damage | Yes, no mortality | No, unadjusted results | Yes, reliable ascertainment of mortality | Yes, 1 year | No, 463/1619 patients not included | 6 |
| Patel 2022 | Yes, AS undergoing TAVR | Yes, reliable evaluation of cardiac damage | Yes, reliable evaluation of cardiac damage | Yes, no mortality | Yes, adjusted results | Yes, reliable ascertainment of mortality | Yes, 2.4 years | Yes, 8/300 patients not included | 9 |
| Pellegrini 2022 | Yes, severe symptomatic AS undergoing TAVR | Yes, reliable evaluation of cardiac damage | Yes, reliable evaluation of cardiac damage | Yes, no mortality | No, unadjusted results | Yes, reliable ascertainment of mortality | Yes, 2 years | No, 222/1063 patients were not included | 6 |
| Schewel 2021 | Yes, severe symptomatic AS undergoing TAVR | Yes, reliable evaluation of cardiac damage | Yes, reliable evaluation of cardiac damage | Yes, no mortality | No, unadjusted results | Yes, reliable ascertainment of mortality | Yes, 2 years | No, 455/1855 patients were not included | 6 |
| Sevilla 2023 | Yes, moderate and severe asymptomatic AS | Yes, reliable evaluation of cardiac damage | Yes, reliable evaluation of cardiac damage | Yes, no mortality | No, unadjusted results | Yes, reliable ascertainment of mortality | Yes, 5 years | Yes, missing data not reported | 7 |
| Shamekhi 2022 | Yes, AS | Yes, reliable evaluation of cardiac damage | Yes, reliable evaluation of cardiac damage | Yes, no mortality | No, unadjusted results | Yes, reliable ascertainment of mortality | Yes, 1 year | Yes, missing data not reported | 7 |
| Snir 2021 | Yes, subgroup of severe AS | Yes, reliable evaluation of cardiac damage | Yes, reliable evaluation of cardiac damage | Yes, no mortality | No, unadjusted results | Yes, reliable ascertainment of mortality | Yes, 1 and 5 years | No, 3851/12013 patients were not included | 6 |
| Tastet 2019 | Yes, moderate to severe asymptomatic AS | Yes, reliable evaluation of cardiac damage | Yes, reliable evaluation of cardiac damage | Yes, no mortality | Yes, adjusted results | Yes, reliable ascertainment of mortality | Yes, 8 years | Yes, missing data not reported | 9 |
| Viva 2023 | Yes, severe AS undergoing TAVR | Yes, reliable evaluation of cardiac damage | Yes, reliable evaluation of cardiac damage | Yes, no mortality | No, unadjusted results | Yes, reliable ascertainment of mortality | Yes, median of 2.9 years | No, 49/139 patients were not included | 6 |
| Vollema 2019 | Yes, severe symptomatic AS | Yes, reliable evaluation of cardiac damage | Yes, reliable evaluation of cardiac damage | Yes, no mortality | No, unadjusted results | Yes, reliable ascertainment of mortality | Yes, 1 year | Yes, missing data not reported | 7 |
| Zhu 2022 | Yes, AS undergoing TAVR | Yes, reliable evaluation of cardiac damage | Yes, reliable evaluation of cardiac damage | Yes, no mortality | Yes, adjusted results | Yes, reliable ascertainment of mortality | Yes, 2 years | Yes, missing data not reported | 9 |

AS: Aortic stenosis, AVR: Aortic valve replacement, SAVR; Surgical aortic valve replacement, TAVR: Transcatheter aortic valve replacement,

**Supplementary data, *Table S2*:** Echocardiographic parameters.

| **Study ID** | **LVEF (%)** | **LV-GLS (%)** | **LAVi (ml/msq)** | **E/E’ ratio** | **PAPS (mmHg)** | **TAPSE (mm)** |
| --- | --- | --- | --- | --- | --- | --- |
| Avvedimento 2021 | Stage 0/1: 62.6±7.9  Stage 2: 58±7.7  Stage 3: 54±13.2  Stage 4: 50.7±13.1 | Stage 0/1: -19.1±1.6  Stage 2: -17.5±2.7  Stage 3: -17±4  Stage 4: -17±4.3 | Stage 0/1: 29.2±2.8  Stage 2: 51±12.8  Stage 3: 55±14.7  Stage 4: 63.6±15.2 | Stage 0/1: 13.3±3.7  Stage 2: 14.7±5.2  Stage 3: 17.2±4.3  Stage 4: 18.8±8.5 | Stage 0/1: 37.6±8  Stage 2: 36.9±7.2  Stage 3: 48.4±12  Stage 4: 50.1±12.1 | Stage 0/1: 22.7±4.3  Stage 2: 21.3±3.4  Stage 3: 21.3±4.3  Stage 4: 17.9±5.3 |
| Belmonte 2023 | Stage 0: 63  Stage 1: 58  Stage 2: 58  Stage 3: 58  Stage 4: 58 | Not reported | Stage 0: 24±9  Stage 1: 26.1±6.4  Stage 2: 41.7±15.3  Stage 3: 48.5±18  Stage 4: 36.2±13.4 | Not reported | Stage 0: 34  Stage 1: 31  Stage 2: 33  Stage 3: 62  Stage 4: 37 | Stage 0: 22  Stage 1: 22  Stage 2: 22  Stage 3: 22  Stage 4: 15 |
| Berkovitch 2020 | Not reported | Not reported | Not reported | Not reported | Not reported | Not reported |
| Fukui 2020 | Stage 0: /  Stage 1: 58.1±10.6  Stage 2: 55.4±11.6  Stage 3: 51.8±14.3  Stage 4: 31.8±13.3 | Not reported | Stage 0: /  Stage 1: 27.5±5.0  Stage 2: 49.6±15.4  Stage 3: 54.3±19.0  Stage 4: 53.0±14.8 | Not reported | Stage 0: /  Stage 1: 36.0±10.6  Stage 2: 37.9±10.9  Stage 3: 61.2±16.4  Stage 4: 54.7±19.2 | Stage 0: /  Stage 1: 19.4  Stage 2: 25.8  Stage 3: 40.1  Stage 4: 82.1 |
| Généreux 2017 | Not reported | Not reported | Not reported | Not reported | Not reported | Not reported |
| Gutierrez-Ortiz 2023 | Stage 0/1: 59.5±9.9  Stage 2: 58.6±9.4  Stage 3: 57.6±10.5  Stage 4: 53.1±11.0 | Stage 0/1: -15.7±4.0  Stage 2: -15.2±3.7  Stage 3: -14.8±4.3  Stage 4: -13.1±4.3 | Stage 0/1: 28.1±4.5  Stage 2: 50.2±17.1  Stage 3: 60.1±18.6  Stage 4: 56.5±56.9 | Stage 0/1: 12.4±4.0  Stage 2: 15.4±5.8  Stage 3: 18.4±7.0  Stage 4: 15.7±6.6 | Stage 0/1: 27.4±7.8  Stage 2: 32.8±10.6  Stage 3: 52.4±16.7  Stage 4: 39.1±15.2 | Stage 0/1: 22.2±3.6  Stage 2: 22.2±3.7  Stage 3: 20.7±3.3  Stage 4: 16.5±3.9 |
| Maeder 2020 | Stage 0: 61±11  Stage 1: 60±9  Stage 2: 54±13  Stage 3: 54±14  Stage 4: 46±14 | Not reported | Not reported | Not reported | Not reported | Stage 0: 24±5  Stage 1: 23±5  Stage 2: 21±5  Stage 3: 18±5  Stage 4: 17±3 |
| Okuno 2021 | Not reported | Not reported | Not reported | Not reported | Not reported | Not reported |
| Patel 2022 | Not reported | Not reported | Not reported | Not reported | Not reported | Not reported |
| Pellegrini 2022 | Not reported | Not reported | Not reported | Not reported | Not reported | Not reported |
| Schewel 2021 | Stage 0: 56.4±9.2  Stage 1: 55.8±9.7  Stage 2: 53.2±11.8  Stage 3: 48.7±13.9  Stage 4: 44.0±14.7 | Not reported | Not reported | Stage 0: 12.7±4.5  Stage 1: 13.5±6.1  Stage 2: 15.0±6.4  Stage 3: 16.6±7.3  Stage 4: 16.6±6.7 | Stage 0: 35.2±9.2  Stage 1: 35.0±9.9  Stage 2: 42.5±10.4  Stage 3: 54.7±15.3  Stage 4: 61.3±15.2 | Stage 0: 21.1±4.7  Stage 1: 19.3±4.6  Stage 2: 19.1±4.6  Stage 3: 16.8±4.6  Stage 4: 15.3±4.1 |
| Sevilla 2023 | Not reported | Not reported | Not reported | Not reported | Not reported | Not reported |
| Shamekhi 2022 | Not reported | Not reported | Not reported | Not reported | Not reported | Not reported |
| Snir 2021 | Not reported | Not reported | Not reported | Not reported | Not reported | Not reported |
| Tastet 2019 | Stage 0: 66±4  Stage 1: 62±7  Stage 2: 63±7  Stage 3: 64±8  Stage 4: 62±8 | Stage 0: 19.4±3.0  Stage 1: 18.5±2.5  Stage 2: 17.5±3.0  Stage 3: 19.1±2.3  Stage 4: 14.9±5.0 | Stage 0: 26±5  Stage 1: 27±5  Stage 2: 45±13  Stage 3: 56±37  Stage 4: 48±16 | Stage 0: 9.4±2.1  Stage 1: 12.5±5.0  Stage 2: 13.9±6.4  Stage 3: 15.5±6.5  Stage 4: 14.6±5.9 | Stage 0: 31±6  Stage 1: 30±7  Stage 2: 34±8  Stage 3: 54±16  Stage 4: 38±13 | Not reported |
| Viva 2023 | Stage 0-2: 56.9±8.2  Stage 3:60.6±7.8  Stage 4:50.8±12.3 | Not reported | Stage 0-2: 46.1±14.9  Stage 3: 65.8±22.0  Stage 4: 56.8±19.6 | Stage 0-2: 16.2±6.7  Stage 3: 19.9±8.6  Stage 4: 15.5±5.4 | Stage 0-2: 36.6±8.4  Stage 3: 63.9±16.9  Stage 4: 49.2±14.8 | Stage 0-2: 21.3±3.0  Stage 3: 17.7±3.0  Stage 4: 14.5±2.7 |
| Vollema 2019 | Stage 0: 62.9±7.0  Stage 1: 57.8±12.0  Stage 2: 55.1±13.4  Stage 3: 46.9±14.9  Stage 4: 41.6±16.1 | Not reported | Stage 0: 24.8±5.9  Stage 1: 26.1±6.1  Stage 2: 50.8±19.1  Stage 3: 60.4±34.3  Stage 4: 57.9±28.2 | Stage 0: 10.8±2.2  Stage 1: 18.0±8.0  Stage 2: 19.8±10.3  Stage 3: 24.2±11.4  Stage 4: 23.3±12.7 | Stage 0: 26.9±8.7  Stage 1: 30.4±8.5  Stage 2: 34.9±10.0  Stage 3: 61.4±14.6  Stage 4: 42.8±16.6 | Stage 0: 22.2±3.3  Stage 1: 21.9±3.5  Stage 2: 21.8±3.6  Stage 3: 20.1±3.6  Stage 4: 13.3±1.9 |
| Zhu 2022 | Not reported | Not reported | Not reported | Not reported | Not reported | Not reported |

LAVi: Left atrial volume index, LVEF: Left ventricular ejection fraction, LV-GLS: Left ventricular global longitudinal strain, PASP: Pulmonary artery systolic pressures, TAPSE: Tricuspid annular systolic excursion.

**Supplementary data, *Table S3*:** Cardiac damage classification, follow-up and major adverse cardiac event outcomes.

| **Study ID** | **Cardiac damage classification used** | **Follow-up** | **Myocardial infarction** | **Stroke** | **Re-hospitalisation** | **Combined major adverse cardiac events** |
| --- | --- | --- | --- | --- | --- | --- |
| Avvedimento 2021 | Généreux classification with modification of left ventricular global longitudinal strain to stage 1 | 1 year | Stage 0/1: 0/23  Stage 2: 2/106  Stage 3: 2/59  Stage 4: 5/74 | Stage 0/1: 0/23  Stage 2: 2/106  Stage 3: 2/59  Stage 4: 3/74 | Any re-hospitalisation:  Stage 0/1: 0/23  Stage 2: 10/106  Stage 3: 4/59  Stage 4: 9/74 | Not reported |
| Belmonte 2023 | Généreux classification | 3 years | Not reported | Not reported | Heart failure readmission for moderate/asymptomatic severe AS:  Stage 0: 0/19  Stage 1: 0/46  Stage 2: 4/61  Stage 3: 4/30  Stage 4: 3/27  Heart failure readmission for symptomatic severe AS:  Stage 0: 1/23  Stage 1: 1/53  Stage 2: 10/99  Stage 3: 4/38  Stage 4: 3/36 | Not reported |
| Berkovitch 2020 | Généreux classification | 1 year | Stage 0: 5/758  Stage 1: 6/769  Stage 2: 6/730  Stage 3: 1/320  Stage 4: 0/31 | Stage 0: 15/758  Stage 1: 21/769  Stage 2: 14/730  Stage 3: 5/320  Stage 4: 1/31 | Not reported | Not reported |
| Fukui 2020 | Généreux classification | 2 years | Not reported | Not reported | Cardiac-cause readmission:  Stage 1 (reference):  Stage 2: 1.30 95% CI 0.83-2.03, p=0.246  Stage 3: 1.84 95% CI 1.13-3.00, p=0.014  Stage 4: 1.49 95% CI 0.73-3.07, p=0.277 | Composite of death and readmissions for cardiovascular causes:  Stage 0: Not reported  Stage 1: 64/93  Stage 2: 312/426  Stage 3: 116/142  Stage 4: 19/28 |
| Généreux 2017 | Généreux classification | 1 year | Not reported | Stage 0: 1/47  Stage 1: 13/212  Stage 2: 71/844  Stage 3: 27/413  Stage 4: 11/145 | Any rehospitalisation: Stage 0: 3/47  Stage 1: 34/212  Stage 2: 128/844  Stage 3: 79/413  Stage 4: 34/145 | Death, rehospitalisation and stroke:  Stage 0: 5/47  Stage 1: 53/212  Stage 2: 236/844  Stage 3: 140/413  Stage 4: 51/145 |
| Gutierrez-Ortiz 2023 | Généreux classification, Okunu classification and authors own classification | 1 year | Not reported | Not reported | Author’s classification and heart failure readmission:  Stage 0: 17/95  Stage 1: 30/166  Stage 2: 8/64  Stage 3: 7/63 | Not reported |
| Maeder 2020 | Généreux classification | 30 days and median of 3.8 years | Not reported | Not reported | Not reported | Not reported |
| Okuno 2021 | Généreux classification | 1 year | Not reported | Not reported | Not reported | Not reported |
| Patel 2022 | Généreux classification | Mean of 2.4 years | Not reported | Not reported | Not reported | Not reported |
| Pellegrini 2022 | Généreux classification | 2 years | Not reported | Not reported | Hospitalisation for congestive heart failure  Stage 0: 0/7  Stage 1: 3/63  Stage 2: 53/532  Stage 3: 34/154  Stage 4: 14/85 | All-cause mortality and hospitalisation for congestive heart failure:  Stage 0: 1/7  Stage 1: 8/63  Stage 2: 128/532  Stage 3: 57/154  Stage 4: 29/85 |
| Schewel 2021 | Généreux classification | 2 years | Not reported | Not reported | Not reported | Not reported |
| Shamekhi 2022 | Généreux classification with modification | 1 year | Not reported | Not reported | Not reported | Not reported |
| Sevilla 2023 | Généreux classification | 5 years | Not reported | Not reported | Not reported | Not reported |
| Snir 2021 | Généreux classification | 1 and 5 years | Not reported | Not reported | Not reported | Not reported |
| Tastet 2019 | Généreux classification with modification | 8 years | Not reported | Not reported | Not reported | Not reported |
| Viva 2023 | Généreux classification | Median 2.9 years | Stage 0-2: 0/41  Stage 3: 0/10  Stage 4: 3/39 | Stage 0-2: 7/41  Stage 3: 2/10  Stage 4: 1/39 | Cardiac-related hospitalisation  Stage 0-2: 13/41  Stage 3: 4/10  Stage 4: 12/39 | Not reported |
| Vollema 2019 | Généreux classification | 1 year | Not reported | Stage 0: 11/97  Stage 1: 25/282  Stage 2: 58/588  Stage 3: 10/82  Stage 4: 24/140 | Cardiac-related hospitalisation:  Stage 0: 12/97  Stage 1: 46/282  Stage 2: 131/588  Stage 3: 20/82  Stage 4: 25/140 | All-cause death, any stroke and cardiac re-hospitalisation:  Stage 0: 39/97  Stage 1: 128/282  Stage 2: 303/588  Stage 3: 54/82  Stage 4: 93/140 |
| Zhu 2022 | Généreux classification | 1 month | Stage 0: 2/199  Stage 1: 0/51  Stage 2: 1/67  Stage 3: 0/59  Stage 4: 0/51 | Stage 0: 5/199  Stage 1: 1/51  Stage 2: 1/67  Stage 3: 2/59  Stage 4: 1/51 | Not reported | Not reported |
